# The fall of the innovation empire and its possible rise through open science

**DOI:** 10.1016/j.respol.2021.104226

**Published:** 2021-06

**Authors:** E. Richard Gold

**Affiliations:** McGill University, Faculty of Law and Faculty of Medicine, Canada

**Keywords:** Innovation, Research productivity, Open science, Intellectual property, Patents, Research incentives, Public-private partnerships, Networks

## Abstract

There is growing concern that the innovation system's ability to create wealth and attain social benefit is declining in effectiveness. This article explores the reasons for this decline and suggests a structure, the open science partnership, as one mechanism through which to slow down or reverse this decline. The article examines the empirical literature of the last century to document the decline. This literature suggests that the cost of research and innovation is increasing exponentially, that researcher productivity is declining, and, third, that these two phenomena have led to an overall flat or declining level of innovation productivity. The article then turns to three explanations for the decline – the growing complexity of science, a mismatch of incentives, and a balkanization of knowledge. Finally, the article explores the role that open science partnerships – public-private partnerships based on open access publications, open data and materials, and the avoidance of restrictive forms of intellectual property – can play in increasing the efficiency of the innovation system.

## Highlights

• Innovations systems have become increasingly inefficient over the last century.
• Complexity, poor incentives and balkanization of knowledge are partly responsible.
• Open science partnerships (OSPs) are one mechanism to reverse declining efficiency.
• OSPs are public-private partnerships that openly share publications, data and materials.
• OSPs avoid restrictive forms of intellectual property to facilitate use and sharing.

## 1 Introduction

Amidst the hype over innovation's contributions to wellbeing and the economy lies growing concern over the declining effectiveness of the innovation system to create wealth and to attain greater social benefit. Despite mounting evidence of this decline, alarm over it nevertheless remains in the shadows of the innovation literature, finding instead a modest voice in environmental sustainability journals (Tainter et al., 2017), among complexity theorists (Youn et al., 2015), some economists (Bhattacharya and Packalen, 2020; Bloom et al., 2020; Gordon 2016; Strumsky et al., 2010) and jurists (Feldman and Wang, 2017). In this article, I document this decline in innovation and the failure of the institutions of innovation to provide incentives and structures that increase the efficiency of the innovation system. Among the reasons for this decline, I examine the growing disequilibrium between proprietary and open science models of innovation that has upset the historical synergy between the two (David, 2008). In the last part of this article, I investigate the potential of open science partnerships to help restore this balance and, through it, to increase the effectiveness of the innovation system.

In Part 2, I describe the empirical literature highlighting three aspects of innovative activity over the past century, using the US as the focus of this study. First, I canvass the evidence supporting the proposition that the cost of research and innovation is not only increasing but has been increasing exponentially over the last century. Second, researcher productivity – researcher output, whether measured in papers, lifespan added, or increased agricultural productivity – has suffered a steady decline for over a century. Third, for most of the 20th century, increased investments in science and technology – particularly investments in hiring new researchers – have offset the productivity decline, leading to steady rates in the production of new innovation artifacts such as papers, patents, and products (C. I. Jones, 2002). These investments are occuring, however, at a rate that is unsustainable in the long term.

Despite the overall steady state in research output (at exponentially increasing cost), innovation is having a decreasing economic impact: while economic growth due to innovation increased in the first half of the 20th century, it has been in decline since the 1970s, with only a short respite in the mid-1990s to early 2000s (Gordon 2016; Scherer 1984; Griliches 1998; Field 2009; Alexander 2006). The phenomena of rising costs, reduced researcher productivity, and the maintenance of an equilibrium between the two correlate with a lessening in the novelty of innovation – the degree to which new products and services constitute breakthroughs as opposed to making slight alterations – since the 1950s (Clancy 2017; Krieger et al., 2018; Arora et al., 2015; Wang et al., 2017; Stoeger et al., 2018; Akcigit et al., 2013). While the correlation is likely no coincidence, the direction of causality needs further exploration.

In Part 3, I examine three explanations for the decline. First among these is the observation that science is becoming increasingly hard as we probe ever deeper into nature's secrets. Each new question posed, under this explaination, requires more resources to solve than did the last. Second, researchers and firms are becoming less productive due to scientists and firms being motivated by incentives that push them away from risk-taking and, hence, breakthroughs. The third explanation is based on an increasing disequilibrium between open science – in which knowledge quickly spreads with few limitations – and proprietary science that relies on exclusive rights to keep all or most others out. The historical equilibrium that brought the scientific revolution of the 17th century is now, so the argument goes, in jeopardy, decreasing the efficiency of the innovation system.

In Part 4, I explore the need to revise our innovation system to avoid, as West (2017) suggests, an otherwise inevitable decline in productivity, growth and attainment of social benefit through innovation. In the past, societies have transformed their innovation systems – from the first, to the second, to the third industrial revolutions in which they moved from craft inventor, to industrial laboratory, to scientific (usually university) laboratory (Bruland and Mowery, 2006) – to address new challenges. The next evolution, that I explore in Part 4, may well be a move toward societal innovation in which a diverse set of actors collaborate – in what Levi-Faur (2009) calls ‘regulatory capitalism’ – to take on risk and quickly translate knowledge into innovations (Drahos, 2017). The particular mechanism I explore is the open science partnership.

Open science partnerships (OSPs) are private-public collaborations that have certain common elements: open access publications, open sharing of data, tools and materials and the absence of intellectual property rights that restrict improvement or use of jointly created inventions. OSPs differ from open innovation models that attain openness through selective licensing of exclusive intellectual property rights (Chesbrough, 2019) in both the scope of openness and the transactions costs involved. While one form of open innovation – open-source software – is very open, it is the exception and not the rule (Beck et al., 2020); most open innovation models share knowledge among a much narrower set of actors. In contrast, OSPs engage in sweeping openness of explicit knowledge, reducing costs of sharing knowledge and of entering into contracts, while providing private value to firms through privileged access to tacit knowledge that expands value, increased reputation and marketing, and by de-risking product and service development. That is, contrary to Chesborough, OSPs do not exist only in the realm of science: they directly “promote the application of that science in the commercial realm” (Chesbrough, 2019, 55).

## 2 Increasing costs and declining productivity: the unsustainable research enterprise

### 2.1 Increasing costs

For the last 100 years, the costs of research and development have increased exponentially, whether measured in expenditures – salaries, equipment, and overhead – per technical person or the overall costs of research and development. We examine the data here.

In the early 1960s, observers began noticing the rapidly rising costs of research and development per researcher (Wolfle, 1960). Machlup (1962) found a three-fold increase in expenditures per technical person between 1941 and 1958 while Milton (1966) found a five-fold increase in these expenditures in the period between 1920 and 1964. In his 1963 book, de Solla Price calculated that the number of scientists had been growing exponentially since the 17th century. He suggested that this rate of growth was not sustainable in the longer term, as “exponential growth eventually reaches some limit, at which the process must slacken and stop before reaching absurdity” (de Solla Price 1986, 18).

**Fig. 1:**
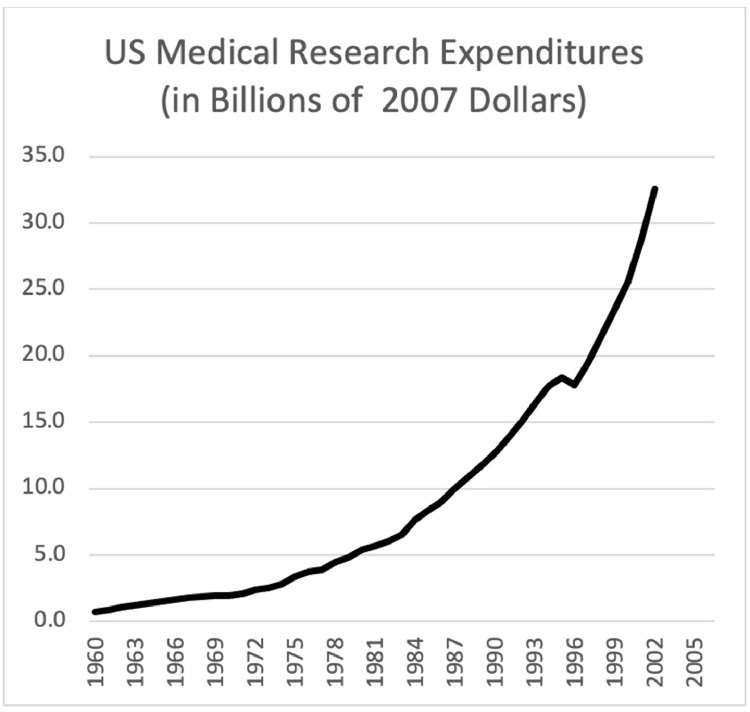
Medical research expenditures 1960–2006 in Billions of 2017 US Dollars. From Statistical Abstract of the United States: 2010 (129th Edition), Table 127, National Health Expenditures–Summary, and Projections.

The data confirm that since 1965, US research expenditures continued to rise. Between 1981 and 2016, these expenditures tripled.^1^ The federal government's research investments similarly rose: across all fields by a factor of just over 1.7 between 1965 and 2006^2^ and, with respect to medical research alone, by a factor of 4.4^3^ over the same period. The number of STEM workers in the US quadrupled between 1965 and 2011.^4^ As Fig. 1 illustrates, medical research expenditures per person working in the life sciences saw a nine-fold increase between 1960 and 2000. DiMasi et al. (2016) have collected data on the cost of a large pharmaceutical firm bringing a single new molecule to market since the 1970s. While there is some controversy over whether they overstate those costs, they have employed the same method throughout the decades. Their data shows exponentially increasing costs in bringing a single molecule to market. While the price of a drug may not correlate with the costs of innovating it, data from Bach (2018) reveal that the median cost of cancer drugs is also rising exponentially.

### 2.2 Decreasing productivity

While Milton (1966, 15) assumed that research productivity per technical person increased at the same time as did costs – “[t]he augmentation by machines, for example, has increased the productivity of the average technical man-year to an unmeasured degree” – this turned out not to be the case. Rates of research and innovation productivity – investments, patents, papers and innovations per technical person as well as health, agricultural and other gains per paper and invention – declined even while investments increased. As Rescher (1978, 87) summarized, “the rapidly – indeed exponentially – increasing pace of effort-investment tends to mask the fact that the volume of high-quality returns per *unit* investment is apparently declining.”

Earlier data regarding patent filings illustrated the problem of declining productivity. As early as 1936, Sanders (1936) concluded that, based on data between 1834 and 1934, while the number of patents per capita increased in the transition from an agricultural to an industrial economy, the rate of patenting seemed “to reach a constant level, or even show some drop” once industrialization took hold. Studies in the 1950s and 1960s refined Sanders's analysis by looking at patents against the number of technical workers rather than the entire population. Schmookler (1954) found that, despite an absolute increase in patent applications between 1870 and 1940, the number of patent applications per technical worker declined. Machlup (1962) found a similar decline between 1941 and 1958.

Hausman et al. (1981) determined, based on patent and research and development data from 1968–1974, that firms suffered from a declining ability to translate their R&D investments into patents. Examining a variety of measures of productivity and innovation – GDP, education spending, as well as patents – Huebner (2005, 984) calculated that the US rate of innovation has been declining since 1916. Jones (2002, 220) noted that, despite the fraction of US STEM workers in the population increasing threefold (from 0.25 percent to 0.75 percent) between 1950 and 1993, “the growth rate of U.S. per capita GDP has been surprisingly stable.” Because infinitely increasing the number of STEM workers is unsustainable, he concluded, growth due to technology “must come to an end” (C. I. Jones 2002, 235).

Total factor productivity (TFP) – the principal, if imperfect, measure of the pace of innovation and technical progress – peaked in 1940–1950 and has been steadily declining since, with a slight but short-lived increase between the mid 1990s and mid-2000s (Gordon 2016, 547; Griliches 1998; Field, 2006). Looking at similar data, Boniatu argued that “the U.S. economy seems to have reached its first threshold of mutation – and hence entered a phase of diminishing returns on innovation – in the thirties” (Bonaiuti, 2018, 1806).

Bloom et al. (2020) conducted one of the most comprehensive studies documenting declining productivity since 1965. They compared economic outputs to investments made in research and development at both the macro and micro levels, and found the same phenomenon: research productivity was in systemic decline. At the macro scale, they measured economic output due to innovation in terms of TFP: “We find that research productivity for the aggregate U.S. economy has declined by a factor of 41 since the 1930s, an average decrease of more than 5% per year” (Bloom et al., 2020, 1105). At the micro level, whether measuring productivity in terms of yield rates for agricultural products, new drugs placed on the market, years of life saved from cancer or heart disease per publication or clinical trial, or chip density for computer chips, they uniformly found a drop.

Lest one object that Bloom et al.’s findings only apply to older technologies, in which firms are plumbing the depths of a decreasing potential pool of innovations, Strumsky et al. (2010a, 503) examined new fields of technology, such as solar and wind technology, biotechnology and nanotechnology, where “simpler, basic discoveries can still routinely be made,” yet found a similar decline in productivity as in older fields. Based on their empirical analysis, they concluded that “in industrial economies there may no longer be increasing returns in newer sectors to offset diminishing returns in older ones” (Strumsky et al., 2010, 504).

A recent study by Pammolli et al. (2020) suggests that the pharmaceutical industry has seen increased productivity since the early 2000s. This study used, however, a different measure of productivity than other studies in the field: attrition rates of drugs during clinical trials. While the authors found a drop in attrition rates, this may have been due to changes in the regulatory environment that relied increasingly on surrogate end-points^5^ of dubious value (Chen et al., 2020; Darrow et al., 2020) rather than on a real productivity gain. It is thus difficult to know whether their finding of increased productivity in the pharmaceutical industry is real or is simply a result of regulatory changes.

### 2.3 A divergence over patent data

There is one notable exception in the empirical data on the productivity decline: from 1985 to 2013, the US went through a patent explosion. While patent applications per STEM worker were roughly stable between 1965 and 1985, domestic patent applications per STEM worker almost doubled (1.88)^6^ between 1985 and 2011. In a similar break with history, the number of domestic patent applications per research dollar more than doubled (2.13) between 1985 and 2013.^7^ This large upsurge in patenting led Gordon (2016, 567) to state that “[t]here is no debate about the frenetic pace of innovation activity, particularly in the spheres of digital technology, including robots and artificial intelligence.”

There is, however, good reason to doubt this apparent frenetic pace of innovation between 1985 and 2013 (Gallini 2002). Kortum and Lerner (1999) argued that the patent upsurge was likely due to firms adopting better management or automation of the innovation process rather than increased innovation. Hall (2004) attributed the upsurge to strategic behavior by firms in complex product industries where products depend on multiple and broadly held patents. Rather than acquiring patents to protect key innovations, these players acquired large portfolios of patents “even those of dubious quality, that is, even those that they have no intention of enforcing” to attract venture capital to early-stage firms (Hall, 2004, 18). An empirical study by Danguy et al. (2014, 561) similarly concluded that strategy, rather than innovation, was driving global patent rate increases: “[T]he ‘global patent warming’ that is currently underway is essentially the result of the internationalization of patent applications and not a consequence of increased research productivity.”

As the above summarizes, the patent explosion that began in the 1980s appears more due to a change in intellectual property management strategy than to effiency of the innovation system. Combined with the data on increasing costs and decreasing productivity, the evidence is strong that we are witnessing an innovation system that is growing less effective in creating wealth and social benefit. This decline has consequences, as I next examine: more risk adverse behavior that signals even greater future decline.

### 2.4 Increasing risk adverse research and innovation behavior

Starting in the 1950s, both firms and academic researchers narrowed the scope of their research and innovation efforts, preferring safer rather than more novel innovations (Strumsky et al., 2011). This occurred at approximately the same time as research and innovation costs ratcheted up, leading to the hypothesis that firms faced with increasing costs decided to reduce their risk by taking on less innovative research. Akcigit et al. (2013b, 4) reasoned that more high risk “ideas are costly to pursue, so inventors focus on reuse/refinements.”

On the industrial front, Youn et al. (2015, 6) found that “the proportion of technological combinations (that is, inventions) that are ‘narrow’ began to increase and currently stands at about 50%.” Clancy (2017b) similarly found that “US patents have made increasingly less novel connections among technological constituents since the 1950s.” Similarly, Krieger et al. (2018, 4) documented “a decline in innovativeness of small molecule drugs over time” through their examination of investigational drug databases. Fojo et al. (2014, E7) attribute this decline to a desire to reduce the riskiness of earnings. They concluded that while a breakthrough, if successful, would lead to higher long-term earnings, if this “strategy is so risky that investors lose confidence and sell their shares,” they would suffer a drop in stock price. This complements the finding by Arora et al. (2015, 2, 5) that “large firms are withdrawing from investing in science internally and focusing more on development,” “leaving universities and small firms to generate new ideas.”

On the academic side, Edwards et al. (2011) demonstrate how firms and researchers continued to explore the same limited set of research targets while ignoring most targets. For example, they found that 65% of 2009 publications focused on the same 10% of proteins as had been copiously studied between 1950 and 2002. As a result, they concluded that “[m]uch of the work that has emerged from exploring the human genome over the past ten years lies fallow” (Edwards et al., 2011, 165), a significant inefficiency in the system. Similarly, Stoeger et al. (2018, 7) found that “while biomedical research does focus on important genes, a disproportionally high amount of research effort concentrates on already well-studied genes.” Using machine learning techniques, they determined that this conservative selection of research targets meant that “even highly promising genes that could already be studied by current technologies remain ignored” (Stoeger et al., 2018, 10).

On the other hand, Pammolli et al. (2020) document an increase in the novelty of pharmaceutical innovation based on two factors: the indication for the drug and its mechanism of action (i.e. its biological target). One possible explanation for this result is that declining regulatory standards reduced innovator risk, adjusting their cost-benefit analysis to support their pursuit of higher-risk research. Alternatively, lower regulatory standards may have led to higher cost medicines with no superior efficacy or safety replacing older, less expensive, medicines (Saluja et al., 2018). This would result in more expensive and less effective medicines entering the market, doing little to increase the efficiency of the innovation system.

## 3 Explanations for the decline

The question left open from these observations is why, contrary to Milton's beliefs, research productivity has been declining. The literature offers three explanations for this decline: 1) with time, science becomes more costly, requiring greater investments to produce the same level of result; 2) science and science funding is skewing toward mediocrity, including through a misalignment of incentives for researchers and for firms; and 3) increasing reliance on early-stage, university, patenting has led to a balkanization of efforts. I examine each in turn.

### 3.1 Complexity in science

Rescher (2014) has long argued that science is both more expensive and less productive because the questions we pose are increasingly complex. He reasoned that scientists solved the easy problems early on. As science progressed, the difficulty of extracting knowledge – with an increased need for technology, energy and staff – grew. He concluded that “the increasing resource requirement for digging into ever deeper layers of complexity is such that successive triumphs in our cognitive struggles with nature are only to be gained at an increasingly greater price” (Rescher 2014, 64). Weitzman (1998, 333) agreed, suggesting “that the ultimate limits to growth may lie not so much in our abilities to generate new ideas, as in our abilities to process to fruition an ever-increasing abundance of potentially fruitful ideas.”

B. F. Jones (2009) examined one aspect of this complexity: the ability to absorb and deploy an ever-richer set of scientific knowledge. As science progressed and required greater knowledge, he hypothesized that scientists would deploy a combination of three strategies: 1) individual researchers would need to absorb more knowledge, delaying when they began their careers; 2) researchers would become more specialized; leading to 3) the need for larger teams. Using U.S. inventor data from 1975 to 1999, he found: “an upward trend in team size that is both general and steep”; an average increase of age of first invention of 0.66 years per decade across all fields; and a 6% increase in specialization per decade. Similarly, Levitt and Levitt (2017) found that the age of scientists winning their first grants from the National Institutes of Health increased from about 36 to 44 years between 1980 and 2011.

It is certainly true that some new technologies, such as CRISPR-Cas9 (Doudna and Charpentier, 2014), greatly simplify research and require less expensive technology. Nevertheless, as discussed in 2.2, Strumsky et al. (2010a, 503) found decreasing rates of productivity in new fields generally, including in biotechnology, solar, wind and nanotechnology. Thus, while there are cost-saving new technologies – with even significant savings – the overall trend toward higher costs appears to hold. Following Rescher and others, the problem seems to lie more in the way we organize science and innovation – the institutions, models of organization, use of intellectual property rights, etc. – than the complexity of the questions researchers investigate.

### 3.2 Mediocrity and misalignment

Tainter proposed a second reason for decreasing productivity in the face of increasing costs: that research trends toward mediocre, middle of the road, and non-disruptive science and away from high-risk, breakthrough explorations. Tainter's argument, building on that of de Solla de Solla Price, 1986, 92), was that the average scientist today is of a lesser quality than that of yesterday due to the greater expansion in the number of researchers (Tainter, 1988). Indeed, between 1950 and 1993, C. I. Jones (2002, 220) found that the fraction of STEM researchers in the US tripled. While Tainter argues that this extra mass of researchers dilutes the effect of extraordinary scientists, there is no evidence to support this and seems to buy into a biased understanding of assessing quality (Kaatz et al., 2016; Wang et al., 2017). It further ignores the reality that the era of the lone scientist has given way to team science (B. Uzzi et al., 2013).

Mediocrity comes in various guises, however. To render the concept more objective, and thus tractable, we can interpret mediocrity to mean a trend toward average, rather than exceptional, creativity. The literature on creativity and its component parts has grown over the decades (Amabile, 1983). In particular, Lee et al. (2015) identified two aspects of creativity that apply to scientific outputs: impact and novelty.

A decline in research impact may help explain the cost and productivity problem. As Lee et al. (2015, 695) noted, impact is “realized through a social process interacting with the community and is therefore ultimately an ex post and subjective judgment” of the value of research. With this in mind, we can ask whether the incentives (and discentives) universities and firms establish to encourage teams to innovate lead to less productive outcomes. Specifically, do these incentives lead teams to expend ever more resources to obtain fewer innovations or innovations that offer ever lower productivity gains in health, the environment or the economy?

Assessing real impact – the effect of a journal publication or innovation on changing real world outcomes – is difficult so both universities and firms measure something else: impact factor for universities and patent applications for firms. Neither captures impact fully, setting up perverse incentives.

Universities and funding councils generally assess academic impact through citation analysis (McKiernan et al., 2019), not on the basis of the direct impact an artifact has on health or the economy. Because of the assumption that the more a paper is cited, the more important and, hence, novel it is, universities and funding councils only peripherally assess real impact. Wang et al. (2017, 1417) find, however, that the assumption that impact measures novelty is wrong. They conclude that more novel papers are actually less likely to be published in high Impact Factor journals – journals with a high average number of citations. They attribute this conclusion, in part, to the fact that novel papers take longer – more than 5 years – to achieve a high number of citations. As Journal Impact Factor is calculated on the basis of citations to articles published in that journal over only the previous two years (Garfield, 1999), the calculation ignores the higher long-term impact of novel articles. Given the two-year window for assessing impact, journals focus on publishing papers that generate short-term impact as they obtain no advantage from a paper with only a long-term impact. At the same time, academic researchers focus on publishing papers that generate short-term citations, even at the expense of novelty.

Given how much weight peer review committees place on Journal Impact Factor, Wang et al. (2017, 1425) argue that there is a bias against novelty that applies “not only to funding decisions but to science policy more generally.” Because of this bias, “competitive selection procedures encourage relatively safe projects, which exploit existing knowledge, at the expense of novel projects that explore untested approaches” (Wang et al., 2017, 1416). Bhattacharya and Packalen (2020b, 17) concur, arguing that “[p]eer reviewers—a conservative lot if there ever was one—abet this tendency since grant applicants can credibly reassure them the proposed work is likely to produce visible, if marginal, successes.” Both Rzhetsky et al. (2015, 14,572) and Packalen and Bhattacharya (2018) give empirical support to this argument. Analysing millions of biomedical papers over a 30-year period, Rzhetsky et al. found that most researchers pursue conservative, low-risk, strategies, focusing on well-known molecules and “rarely wander far across the knowledge network or bridge disconnected chemicals.” This is exacerbated by the scarcity of funding opportunities that encourage risk-taking (Azoulay et al., 2011).

Industry also leans towards lower impact research. In the pharmaceutical field, Fojo et al. (2014, E9) argue that “the rapidly rising cost of cancer therapies, the regulations governing their adoption by public and private insurers, and the increasing economic risk of drug development have had the unintended consequence of stifling progress by diverting enormous amounts of time, money, and other resources toward therapeutic indications that are arguably marginal.” More broadly, Strumsky et al. (2011) found that commercially-oriented researchers increasingly turn toward exploiting existing knowledge to generate small improvements rather than undertake riskier research that would expand product development in new directions. They speculate that researchers do so “[u]nder pressure to generate patents in copious amounts” (Strumsky et al., 2011, 8). This was particularly true during the patent explosion that started around 1985, discussed earlier at 2.3. Feldman (2018) documents that, between 2005 and 2015, pharmaceutical firms focused more on protecting past drugs through additional patents than on discovering new medicines. Due to strategic uses of patent law, “there is a complete undermining of the system for pharmaceutical innovation as the repeated addition of protections, one after another, pushes competition further into the future, threatening innovation in the process” (Feldman, 2018, 639).

For both industry and universities, the incentives they provide to encourage impact actually decrease novelty and have little to do with real world impact. There is thus a deep misalignment between incentives and innovation, leading to lower novelty.

### 3.3 Balkanization through university intellectual property

The economics literature is frustratingly in no better position today than it was in the 1950s to answer the question of whether patents increase or decrease overall innovation (William, 2017; Gallini, 2017; Sampat and Williams, 2018; Hall, 2019). Further, there is evidence that, while intellectual property and economic growth are correlated, the direction of causation may be from growth to higher levels of intellectual property protection, mediated by politics, rather than from intellectual property to growth (Morin and Gold, 2014; Gold et al., 2019). We do know that certain industries have constructed themselves around the availability of patents and hence incumbents remain dependent on them (Hall and Harhoff, 2012; Galasso and Schankerman, 2015). These industries include the chemical, pharmaceutical and biopharmaceutical industries. We also know that the availability of patents shapes the fields and nature of innovation, even if their effect on overall levels of innovation is uncertain (Moser, 2013).

We have increasing evidence concerning the effect of university-held patents on innovation, although the literature is not yet conclusive. On the positive side, there are certainly technologies that emerged from universities through patenting into socially valuable innovations (Hockstad et al., 2017; Allard et al., 2018; Reinhart, 2020). Some of these relied on patents as a key instrument used to attain those benefits (Bremer et al., 2009). Further, Walsh et al. (2003) point out, using interview data, that broadly licensed university biotechnology research tools – such as PCR and recombinant DNA methods – impose relatively small extra costs and delays. On the negative side, university patents impose a number of transaction costs, whether through decreased freedom-to-operate (Gaessler et al., 2019) or through increased university patenting – documented by Bremer et al. (2009) – that entails not only the direct costs of obtaining a patent but accompanying litigation and negotiation costs.

One must also be mindful that the benefits of university patenting are tempered by three factors. First, as Williams (2010) demonstrated, increased costs of accessing knowledge decreases the level of follow-on use of that knowledge. Second, the fact that universities used patents as a mechanism to transfer inventions to the private sector does not imply that the private sector could not have obtained the inventions through other mechanisms as efficienly. For example, a firm working in concert with a non-patenting university could develop and patent its own invention based on the collaboration. This is what occurred when Celgene acquired a patent over a drug directly building on previous unpatented research done in collaboration with the Structural Genomics Consortium (“The Ontario Institute for Cancer Research and the Structural Genomics Consortium Develop and Give Away New Drug-like Molecule to Help Crowd-Source Cancer Research” n.d.). Beyond this, universities have under-explored alternative intellectual property regimes – such as regulatory data protection – that provide fewer restrictions on use of the invention than do patents. Third we do not – and may never truly – know the quantity of university-originated innovations that would have come about but never materialized because of lack of freedom to operate, the threat of patent litigation from universities or their licensees (Gold and Carbone, 2010), restrictive licensing, or delays caused by negotiations over patents.

Thus, one needs to temper assertions that the absence of university patents “would inevitably slow the development and reduce the availability of new treatments and vaccines” (Reinhart, 2020) with the reality that the empirical literature is mixed at best. Still, it is quite plausible that, in the absence of university patents, certain technologies would either be delayed or (less plausibly) never developed. On the other hand, the empirical literature also suggests that in the presence of those patents, other technologies are likely delayed or never developed.

It is thus unsurprising that the literature suggests that the move to university-owned and controlled patents, accelerated, in part, through the 1980 Bayh-Dole Act (Mowery et al., 2001), did not demonstrably achieve either of the two overarching goals of the practice: to increase the level of innovation in the economy and to increase revenue gains for universities (Eisenberg and Cook-Deegan, 2018; Ouellette and Tutt, 2020; Corredoira et al., 2019). There are several reasons put forward to explain why a university patenting strategy has not had the desired results, including decreased downstream development and upstream duplication (Egelie et al., 2019), increased difficulty and delays in establishing contractual relationships with university technology transfer offices (Dahlborg et al., 2017; Hertzfeld et al., 2006; Kira R. Fabrizio, 2006), lack of university expertise and market knowledge (Swamidass and Vulasa, 2009), delayed dissemination and uptake of results (Williams, 2013; Fabrizio, 2009; Kira, 2006; West, 2006), perverse university incentive structures (Ouellette and Tutt, 2020; Eisenberg and Cook-Deegan, 2018) and the use of university patents to sue firms that have developed products without the aid of university patents (Eisenberg and Cook-Deegan, 2018, 82; Rooksby, 2011).

Other forms of intellectual property rights, notably trade secrets (Williams, 2013; Gallini, 2017; Sampat and Williams, 2018) and university contractual relations (Walsh et al., 2005) also reduce the subsequent use of knowledge. Secrecy leads to data silos that hamper further research, especially when combined with privacy and informed consent rules (Rai, 2017). Negotiations over intellectual property rights with universities create complexity and thus either delay or result in the failure to reach a deal (Hertzfeld et al., 2006; Kira R. Fabrizio, 2006).

In summary, the argument in favor of Bayh-Dole is mixed at best. There exist reasons to believe that not only do university-held patents, but other forms of intellectual property such as trade secrets, increase the costs of both current research efforts – through delay in establishing research collaborations – and future research. Whatever benefits that may arise from university patenting are likely outweighted by the balkanization of knowledge that they create.

### 3.4 Summary

While none of the three explanations explored above – increased complexity, misaligned incentives, and knowledge silos protected by intellectual property – may alone explain the increasing inefficiency of the innovation system to create wealth and attain socially beneficial innovations, together they threaten the logic of the status quo approach to innovation policy. In the short-term, governments can only maintain current levels of innovation through increasingly large injections of resources.

Meanwhile, at the individual and firm level, actors continue to move away from risk, toward less radical and less productive innovation. Consumers, patients and firms seeking productivity gains through innovation will see declining benefit from them both in terms of quality of life and economic growth. Measures of innovation based on patents and impact factors may rise, but these are illusions caused by strategic behavior rather than increased productivity. With declining economic productivity and declining rates of socially beneficial innovations, at some point governments may no longer be willing to fund research and development. With firms increasingly unwilling to fund the development of the basic knowledge to spur innovation, the result could very well be a further, steeper, decline in the efficiency of the innovation system.

## 4 Innovating the innovation system through open science partnerships

To avoid a steeper decline in the efficiency of the innovation system in creating growth and acheiving social benefit, and perhaps even reverse it, we need to experiment with the institutions that form that system (OECD and The World Bank, 2014). We need a system that generates regular innovations (Abernathy and Clark, 1985) that makes small improvements to existing products and services, cumulative innovation that builds on existing innovations to introduce new products and services to existing or new markets (Galasso and Schankerman, 2015; Scotchmer, 1991; Gallini, 2017), and breakthrough innovations that produce new goods and services that open new markets, cure previously untreatable diseases, and transform the economy (Panagopoulos, 2011). A well-functioning innovation system attends to all these forms of innovation (Lundvall et al., 2002).

Paul David argued that the past success of our innovation system is due to its combination of proprietary and open models of science, held in proper balance (David, 1998; 2003, 19). While proprietary models of science depend on patenting and licensing, open science involves the broad and free sharing of ideas and knowledge without intellectual property restrictions (Gold, 2013). Open science is particularly good at producing new knowledge – because it increases the capacity to build upon knowledge created by others – while proprietary science is stronger at translating the existing stock of knowledge into innovations (Dasgupta and David, 1994, 516, 518; P. A. David, 2008, 21) due to the ability to capture value through intellectual property. The key public policy problem, David (2008, 7) argues, “is to keep the two sub-systems in proper balance by public funding of open science research, and by checking excessive incursions of claims to private property rights over material that would otherwise remain in the public domain of scientific data and information.”

David envisions ‘open science’ as a complement to a property system by establishing an institutional “alternative to the intellectual property approach” of controlling access to scientific knowledge (David, 2003, 19). Open science relies on academic rewards that, despite their flaws, aim at generating knowledge rather than on economic gains. It not only involves the free sharing of research publications (open access) and of data (open data) (Fecher and Friesike, 2014) by relinquishing copyright restrictions, it eliminates restrictions on the use of tools, materials, and processes imposed by patent rights. Researchers build knowledge not because of intellectual property incentives, but because of academic ones such as promotion, the likelihood of obtaining research grants, and reputation. The absence of proprietary control over data, publications and results in open science positions other researchers to push the limits of knowledge even further.

As David explains, open science emerged in late 16th-early 17th century Europe out of an intricate and historically contingent process, not as a solution to failures in the innovation system (David, 2008, 6). It was an efficient solution to the problem of evaluating the quality and importance of research outputs as well as to building new knowledge The benefits of open science on innovation efficiency were nevertheless profound: David argues that it was this historical development that placed Europe, rather than other previous or then-current scientifically advanced civilizations, as the global innovation powerhouse for centuries (David, 2008; Mokyr, 2016). Policy changes over the past several decades have, however, diminished the role of open science in favor of balkanized and proprietary science, upsetting the critical balance between the two (David, 2008; 2014).

The imbalance between proprietary and open systems of scientific and technological production contributes to many of the previously explored inefficiencies and problems in today's innovation system. Costs rise and efficiency decreases when knowledge useful for further innovation is not shared or is surrounded by legal barriers (Galasso and Schankerman, 2015; Williams, 2013). Gaessler et al. (2019) show that patent thickets and fences block follow-on innovation by reducing freedom-to-operate, even in discrete technology fields. Bringing together the diversity of skills, data and knowledge needed to address complex scientific questions is more difficult in the face of extended negotiations over proprietary rights and siloed knowledge (Hertzfeld et al., 2006). Breakthroughs occur less frequently when it becomes burdensome to construct teams that include new players (Uzzi and Spiro, 2005) who bring new perspectives and knowledge. As David (2008, 4) pointedly remarks, it is not a question of a preference for proprietary or open systems, but how they work together “so that the special capabilities of each may amplify the productivity of the other,” whether that be in parallel or in series.

With the aim of returning to an equilibrium between proprietary and open approaches to innovation, I introduce a model of open science that specifically aims at addressing the ills discussed in Section 2 of increasing costs, decreasing levels of risk-taking, and balkanization that together lead to declining productivity: open science partnerships (Gold, 2016). These are goal-oriented collaborations undertaken by a mixture of private and public sector actors that are diverse and that openly share knowledge, data and other artifacts without the use of intellectual property rights that prevent others from using or building upon those artifacts. Their aim is to increase knowledge creation, development and transfer, accelerate innovation, decrease transaction costs, and promote closer to frictionless tacit knowledge transfer (Ali-Khan, Jean, and Gold, 2018; Gold et al., 2019). Significantly, these open science partnerships lead not only to value creation but, contrary to Chesbrough (2019), to the ability to capture value even in the absence of traditional forms of intellectual property.

Two longstanding examples illustrate the nature and structure of OSPs. The Mario Negri Research Institute, with three locations across Italy, became the first biomedical open science establishment in 1961, pursuing basic and clinical research across a number of medical fields without patents (Light and Maturo, 2015). Its work is supported by a mixture of public and private funding (“How We Support Ourselves |Mario Negri” n.d.). The Structural Genomics Consortium, with campuses across the globe, is an OSP that conducts basic research, supports clinical trials and makes data and materials available without the use of patents. It consists of academic researchers and pharmaceutical firms funded by philanthropy, the private sector and government agencies (Morgan Jones et al. 2014b; Perkmann and Schildt 2015). Both are charities.

OSPs differ from other forms of open science (Fecher and Friesike, 2014) in important ways. General open science initiatives focus on both rendering publications, data and peer review open by removing copyright barriers and providing the infrastructure through which they can be shared and measured. Fecher and Friesike call the former a combination of the ‘public school’ and ‘democratic school’ of open science and the latter a combination of the ‘infrastructure school’ and the ‘measurement school.’ Each of these approaches aims to make research more transparent and responsive to the public but do not, in themselves, change the incentives or overall productivity of the innovation system. A fifth approach – the ‘pragmatic school’ – comes closer to this goal by focusing on how the open sharing of knowledge can facilitate the modularisation of science and collaboration through online tools.

By contrast with these other approaches to open science, OSPs aim to restructure the relationship between partners within defined research collaborations by combining different forms of incentives (Fabrizio, 2009; Perkmann and Schildt, 2015) and by removing roadblocks not only to the sharing of information, but to its use. OSPs openly share, with only minimal restriction (usually related to patient or user confidentiality, informed consent, and other regulatory obligations), data, tools and materials with the global research community without the exclusivity provided by patents or copyrights. Within its network of partners, OSPs create relationships of trust (Light and Maturo, 2015) to support tacit knowledge exchange through formal and informal mechanisms within the partnership (Morgan Jones et al., 2014a). Through these tacit knowledge exchanges, partners are able to develop products and services significantly earlier than are their competitors.

A case in point introduced in Section 3.3 is the development of a drug against leukemia developed through an open science partnership between the Structural Genomics Consortium and the Ontario Institute for Cancer Research that not only resulted in the largest pre-clinical drug deal in Canadian history, but shaved off two or more years from expected development times (“The Ontario Institute for Cancer Research and the Structural Genomics Consortium Develop and Give Away New Drug-like Molecule to Help Crowd-Source Cancer Research” n.d.; Gold and Morgan, 2019). Because tacit knowledge is more difficult to transfer over distance (Von Hippel, 2005), OSPs tend to also promote local high quality employment. These benefits appear to be long-lasting (Bloom et al., 2020)

These characteristics of OSPs address the three underlying factors explored earlier at 2.5 to explain the productivity decline and increasing costs in the innovation system: mounting complexity in science, lack of incentives to take on risk, and the balkanization of knowledge through intellectual property. I discuss each in turn.

### 4.1 Addressing complexity

While the complexity involved in unraveling nature's secrets shows no signs of abating, OSPs offer a mechanism to manage that complexity by: (i) creating conditions under which teams are more likely to be diverse, and (ii) ensuring the better flow of knowledge among actors within and outside of the partnership. Both of these mechanisms promise to increase the efficiency of the innovation system.

OSPs create an environment that remove barriers to ensure that teams are diverse. They do so by reducing or removing transaction costs involved in establishing teams, particularly when universities are involved, through the elimination of protracted negotiations, principally with respect to intellectual property (Hertzfeld et al., 2006, 826; Dahlborg et al., 2017), and the use of standard form agreements that set out general terms of the collaboration (Rai et al., 2009; Kieff, 2005; Roskams-Edris and Gold, 2019). The reduction of these costs creates conditions under which it becomes viable for smaller firms, firms that are more tangential to the core project (e.g., an artificial intelligence firm in drug discovery), and user or patient organizations, to participate in the partnerships.

There is strong overall evidence that diverse teams are more innovative than are homogeneous ones (Hong and Page, 2004). Diversity can take different forms. For example, both Kerr (2013) and Hunt (2011) document the disproportionate contribution that non-European immigrants make to U.S. innovation. Hong and Page (2004) show that increases in functional diversity – differences in training and education – lead to greater innovation, even if individual skill levels are the same or lower than in less functionally diverse teams – albeit at the risk of increased interpersonal tensions. Sampson (2007) finds benefits from a moderate level of technological diversity: moderately diverse partnerships are more likely to innovate than are those with either a low or high level of technological diversity. A third form of diversity is the degree to which team members have previously worked with one another (Fershtman and Gandal, 2011). Uzzi and Spiro (2005) suggest that creative teams consisting of a combination of individuals with existing relationships with new members within a small world network structure are more successful than are teams consisting primarily of old colleagues or mostly new members. Too large a proportion of those having previous relationships leads to stagnation while too few preexisting relationships leads to a lack of cohesion and lower trust.

A dissenting article by Carayol and Maublanc (2018) argues that greater interdisciplinarity on research teams decreases the impact of their publications. As noted in 3.2, impact is different from novelty and thus declining impact may not indicate reduced novelty. A closer look at Carayol and Maublanc (2018) indicates, in fact, that the authors measure impact through citations over the short-term – over a three year period – rather than the long-term when interdisciplinary publications are likely to have a larger rate of citations. As discussed in 3.2, more novel papers take over 5 years to reach impact because of their diffusion across a larger variety of disciplines. Thus, nothing in Carayol and Maublanc (2018) undermines the expectation that interdisciplinary publications have a lower impact than do single-discipline articles over the short term but that, over a longer term, they have a greater impact.

Overall, teams that combine old partners with new and that possess both identity and functional diversity are best placed to develop novel innovations. At the same time, while teams need to be sufficiently large to be diverse, Lee et al. (2015) find that overly large teams are less likely to produce novel results. They suggest that as teams grow larger, they experience diseconomies of scale: “lower consensus, higher coordination costs, more free-riding, more emotional conflicts, and lower quality of group experience” (Lee et al., 2015, 686).

Because they eliminate intellectual property within the partnership, OSPs remove burdens on the sharing of explicit and tacit knowledge between partners. They accomplish this by putting all publications, data, materials and tools in the public domain, available to all, eliminating intellectual property barriers to internships, visitorships, and the exchange of personnel between partners, ensuring relationships of trust (Lundvall et al., 2002; Chen and Guan, 2010). This removes a major barrier to graduate students and post-doctoral fellows moving from academic to industrial laboratories, as they will face few restrictions on the future publication of results. Several studies found that teams that facilitate knowledge exchange between members, especially of tacit knowledge, are more successful (Arthur, 2007; Rzhetsky et al., 2015; Schoenmakers and Duysters, 2010; Mokyr, 2016; 2011; Björk and Magnusson, 2009).

Dasgupta and David (1994, 503) explain that “[i]t is obviously advantageous to belong to a coalition among whom information will be pooled, because that will give the coalition members a better chance of quickly acquiring all parts of the puzzle and being the first to send it in for publication.” Levin and Cross (2004, 1477) add, “people prefer to turn to other people rather than documents for information.”

By drawing on academic and industry knowledge, OSPs provide a platform to create geographic technology hubs. In her study, Fabrizio (2006) found that firms that are located in proximity to their university partners and possess internal research capacity – in order to assimulate and exploit knowledge obtained – gain the most from working with university partners. Through a combination of three types of empirical studies, Bikard and Marx (2019) show that hubs are several times more successful at translating knowledge – measured as patents citing academic papers emerging from the hub – than in the absence of a hub.

While open science approaches typically involve large numbers of people and firms who share data and publications without barriers to access, OSPs share more openly that other open science approaches while being targetted in terms of their operation. . They are more open in that they not only facilitate the exchange of explicit knowledge as do other open science approaches, but their governance and organization encourage their members – partners – to share tacit knowledge. They are more targeted than other open science models in that they share knowledge with the aim of achieving a clear goal, be it new health interventions, new mechanical devices, new information technology solutions, and involve a core network of members that carry out the work. These networks, being diverse at least in terms of function, technology and previous relationships, can be sufficiently small so as to avoid a lack of coherence.

### 4.2 Incentives and risk

By increasing the diversity of actors, especially by including actors from different sectors, OSPs bring into play a greater range of goals and incentives to motivate innovation (Ali-Khan, Jean, and Gold, 2018; Vickers et al., 2017). Teams that draw on multiple incentives are better able to weather changing financial and political environments than are those that rely on only one form of incentive, often patent rights. The marshaling of reputation (Dahlander and Wallin, 2020; Florko, 2020), population health concerns, patient views, macro-economic wellbeing and profit in the fight against COVID-19 illustrates the complementarity of innovation incentives (Apuzzo and Kirkpatrick, 2020). A team able to combine traditional academic incentives, such as being first to publish, philanthropic incentives – such as addressing social problems –, government incentives to provide jobs and economic growth, and private sector incentives of near-term and long-term profit (Rzhetsky et al., 2015) can not only better withstand a change in the interest of any one sector but can increase diversity of approaches and skills.

Open science teams draw on broad sets of incentives to undertake breakthrough innovation, but only if those incentives are in alignment (Rzhetsky et al., 2015). These incentives include both those that are positive – priority for discovery, access to equipment and research staff, funding, rewards for sharing and partnerships, market exclusivity, fiscal advantages, accelerated regulatory assessment, and so on – or the removal of barriers – such as promotion rules and funding requirements that require patented outputs, a focus on short-term impact factor, intolerance for failure, etc. Governments can support the alignment of these incentives through regulatory advantages for those who share, funding calls specific to open science, fiscal policy to support OSP participation, and intellectual property laws that set high standards on patentability so as to ensure freedom to operate. Together, these initiatives better align private, academic and public incentives to achieve successful outcomes (Eisner, 2007, 282). Some of these may be specific to OSPs – such as funding competition rules and fiscal advantages – and some more general to open science practices.

OSP teams offer a mix of skills and knowledge to undertake breakthrough innovation (Rzhetsky et al., 2015, 14,573). As Munos and Chin (2011) note in respect of biomedical innovation: “Innovation networks offers a better potential to mitigate risk because drug companies can work with numerous partners to reengage in breakthrough research and explore novel hypotheses on a scale that dwarfs what is possible in a single company.” Munos and Chin further argue that working in collaboration with multiple other entities – public and private – lowers the cost of innovation for all, thus lowering overall risk.

Successful innovation networks quickly learn from their failures and build upon their successes (Yin et al., 2019). By drawing on multiple incentive structures and skill sets within environments of trust, OSPs can rapidly validate results by members not directly involved in the original research. Unlike open innovation models, OSPs avoid barriers on knowledge uptake imposed by intellectual property. Through the open validation of results that would otherwise be unlikely in a proprietary setting, OSPs are better positioned to achieve high quality results. For example, when the Ontario Institute for Cancer Research originally developed a probe that underlay its later development of a first-in-class leukemia drug, the partnership was able to quickly assess the probe before sharing it (Gold, 2019). Experts outside the original team found it wanting in some respects, resulting in the Institute developing a new, higher-quality, probe rapidly.

By combining functional and technical diversity – but not too much – OSPs are poised to build on past failures while retaining successful responses. The structured nature of the sharing within networks – as opposed to broad and open sharing with all – create environments that bring new ideas while retaining what has worked (Fleming, 2001). There is a danger that constant sharing of data, ideas and tools can lead to group-think. OSPs are well structured to resist this through their diversity of actors and incentives (Boudreau and Lakhani, 2015, 17).

### 4.3 Avoiding knowledge silos

Beyond managing complexity and increasing diversity, OSPs position team members to overcome barriers to knowledge sharing and reuse by eliminating intellectual property barriers (Ali-Khan, Jean, and Gold, 2018). The Mario Negri Institute adopted its open science policy specifically to remove these barriers. Concluding that “[p]atents are an obstacle to collaboration and an incentive to hide data or methods,” the Institute's founding Director, Dr. Silvio Garattini, led the organization to refrain from holding any patents (Light and Maturo, 2015, 12). This increased trust that, in turn, opened the door to collaboration with firms and academics alike because “pharmaceutical companies have been willing to share advanced work with Institute scientists, knowing it is in safe hands of researchers interested in sharing and collaborating” due to the lack of Institute interest in patents (Light and Maturo, 2015, 14). Morgan Jones et al. (2014) similarly found that the lack of patents encouraged partnerships and accelerated the work of the Structural Genomics Consortium.

Universities, colleges and research institutes have demonstrated no particular skill at managing intellectual property (Egelie et al., 2019). Thus, the lack of patents within OSPs – in which universities and research institutes are important players – comes at little cost. Simply put, few such institutions patent the right things for the right reasons in the right way at the right time (Hertzfeld et al., 2006). In contrast, universities, colleges and institutes have a comparative advantage in three areas of innovation: knowledge creation, knowledge diffusion and being brokers able to bring actors from different sectors together. Rather than create silos of knowledge through intellectual property, universities, colleges, and research institutions would do better by convening public, private and community leaders, and sharing knowledge while relying on an improved academic incentive system to drive knowledge creation (David, 2003, 22). It may be true that the latter is imperfect, relying too heavily on short-term impact factors, but it has a long history of advancing knowledge.

Sharing knowledge, materials, and tools increases quality, speeds validation and reduces duplication of innovation (David, 2003, 22; Egelie et al., 2019; Kira R. Fabrizio, 2006). In contrast, university patenting leads to delays due to protracted and difficult negotiations: “[T]he transfer of technology through the vehicle of licensing intellectual property is, in the case of process technologies, far more subject to tensions and deficiencies arising from the absence of complete alignment in the interest of the involved individuals and organizations” (David, 2003, 26). Patenting comes in later, when a firm builds on the OSP's research and innovations to build and market its own products. The goal is to leave the direct OSP outputs in the public domain, free from patents that prevent others from drawing on the knowledge to build competitive products, while promoting proprietary approaches outside of these. Depending on the technological field and the interdependency of inventions, the firm would either maintain exclusivity over the invention – such as the development of a drug through a modification of the public domain molecule – or licence back the invention to the OSP for use within the scope of the OSP's mandate. As the first to bring a breakthrough product to market is often not the one that profits most (Baba and Walsh, 2010), the OSP's non-patent stance complements and enables a partner firm to later patent and develop markets.

The literature reveals two perceived barriers to broad firm participation in OSPs, thus threatening the efficiency gains they promise: free-riding (West, 2006) and the lack of appropriate incentives (Morgan Jones et al. 2014b; Perkmann and Schildt, 2015). As I have defined them, OSPs not only openly share research results but avoid patenting them, raising the question of whether firms will invest in them when they can, ostensibly, simply use the results without cost. Second, given that firms participate in research in order to gain some advantage in the market, what advantages can OSPs bring that a firm cannot otherwise access?

Existing OSPs have not only succeeded in attracting private sector investment, many OSPs originated with firms. Both the Structural Genomics Consortium and the SNP Consortium (Sachidanandam et al., 2001), for example, arose from industry (Perkmann and Schildt, 2015). Partners derive a variety of benefits even in the absence of intellectual property capture and even at the risk of free-ridiung (Levine and Prietula, 2014). These include value creation and growing the size of the market (West, 2006; Olk and West, 2020), access to complementary, yet uncodified, knowledge, advanced access to knowledge, and simplifying intellectual property landscapes (Kira R. Fabrizio, 2006; 2009), recruiting and retaining scientists – often at lower salaries due to the ability to publish – and marketing (Simeth and Raffo, 2013). As David (2003, 25) concluded, firms place value on access to uncodified knowledge. OSPs offer this access, including through direct participation in joint projects, training and workshops and through the exchange of students and staff between institutions. As Nelson (2001) noted, it is a ‘myth’ that intellectual property is needed.

Introducing more OSPs into innovation systems offers one strategy to return to an equilibrium between proprietary and open models of innovation needed to return efficiency to the innovation system. OSPs add this efficiency by decreasing transaction costs (Nelson, 2001; Kira R. Fabrizio, 2006; West, 2006), speeding up research, providing access to diverse and tacit knowledge, and deploying a set of overlapping and complementary incentives to de-risk research. Industry partners are better positioned than outsiders – even if those outsiders have access to codified knowledge – to exploit OSP outputs by not only leveraging their relationships with partners but through their priviledged access to the direction of knowledge production and to tacit knowledge. In addition, through increased sharing and a commitment not to patent in areas of joint research, OSPs provide the opportunity to shrink patent thickets and fences that reduce freedom-to-operate (Gaessler et al., 2019) A key advantage of OSPs is their ability to accelerate innovation (Morgan Jones et al., 2014b) by fast-tracking project start-up due to lower upfront negotiations over intellectual property and the rapid sharing of tacit knowledge.

Adding more OSPs to the innovation system will help address the complexification of science, the misalignment of incentives and the balkanization of knowledge. Those OSPs that do exist have succeeded in creating substantial trust between private, public and community organizations, in decreasing duplication, and in better ensuring the reproducibility of results (Morgan Jones et al., 2014b), all without patents.

## 5 Conclusion

Studies deploying different methods, measuring innovation through different means and over different time periods, collectively provide consistent and persuasive evidence that the current innovation system is becoming increasingly inefficient and unproductive. While intellectual property and other exclusive rights are critical to continued firm investment in research and development, too much intellectual property actually decreases efficiency through siloing and high transaction costs. Firms retrench to lower-risk projects. The consequence is a declining level of growth due to innovation, rending the argument that governments ought to invest more in innovation precarious. As a result, governments curtail funding, leading university researchers to become increasingly risk-adverse, particularly when facing a peer review system that rewards short term citations over long-term contribution. At some point, as West (2017) speculates, inefficiency leads to more loss of productivity that, in turn, leads to greater declines in efficiency.

Whether the innovation system reaches such a decline in efficiency depends, in part, on whether the actors in the innovation system can curtail cost escalation and increase productivity. Following David (2003), we can fix some of these difficulties by re-establishing an equilibrium between proprietary and open science models of innovation. The last several decades witnessed a slide away from open science toward proprietary models, upsetting a balance that has sustained science for three centuries. The belief that science was only done in the academy and commercialization in industry (Chesbrough, 2019) is not only factually incorrect – the vaccine to fight Ebola was created, developed and trialed solely by the public sector (Herder et al., 2020) and the largest pre-clinical drug deal in Canadian history arose out of an OSP (“The Ontario Institute for Cancer Research and the Structural Genomics Consortium Develop and Give Away New Drug-like Molecule to Help Crowd-Source Cancer Research” n.d.) – but blinds us to advantages of open science partnerships in accelerating commercial development.

The Structural Genomics Consortium transitioned from science to commercial development through novel approaches to intellectual property (Ali-Khan et al., 2018), including using regulatory data protection while fully sharing all innovation artifacts, attracting significant industrial, governmental and philanthropic investment. The consortium's small world network structure positioned it to draw on a diverse set of skills to quickly validate and improve results. As a consequence, industry accepts the consortium's results without the need for further reproducibility studies, a rarity in the pharmaceutical sector.

There remain significant unanswered questions concerning the role of OSPs in overcoming the deficits in the existing innovation system. Many OSPs to date have been in the biomedical/biopharmaceutical field where innovation is complex, large data sets indispensable, and regulatory requirements high. Similar circumstances exist in other technological fields, such as green tech and artificial intelligence, but not in others. More informal open science relationships – outside of a formal partnership – exist in the information technology industry (West, 2003; Levine and Prietula, 2014). Further study of the use of OSPs in different technology fields is required to determine the broader application of these partnerships in overcoming declining productivity.

A second set of questions surrounds the long-term sustainability of OSPs. Without some form of intellectual property, venture capital is not likely to invest in them. Either OSPs will need to engage with some forms of intellectual property, even if not patents, or will need to find other sources of funding. In the biomedical field, OSPs can use regulatory data protection. While shorter in length than patents, they offer superior protection in many ways, including when they begin (at market approval rather than at invention) and the fact that they are difficult to invalidate. Spinouts of the Structural Genomics Consortium rely on this form of protection and have obtained private funding. Not all technological fields benefit from the same variety of intellectual property regimes as does biomedicine. How OSPs will attract funding in these areas remains an open question.

Third, governments interested in encouraging OSPs will almost certainly need to engage in policy reform (Ali-Khan, Jean, and Gold, 2018). This can take the form of advantages offered to OSPs, for example, research grants, regulatory regimes, purchasing and procurement, the provision of a sharing infrastructure, or direct funding. More research is needed to identify policy options and to assess their impact.

As a result of the COVID-19 pandemic, researchers and firms quickly adopted open science to accelerate research and innovation on both characterizing the SARS-CoV-2 virus and developing diagnostics and vaccines. The success of these efforts points to the important contribution that open science can make to productivity. OSPs provide a leading model of how to make this change permanent.

## Author statement

Gold conceptualized, formally analysed, validated, wrote and reviewed the article and acquired funding.

## Declaration of Competing Interest

The authors declare that they have no known competing financial interests or personal relationships that could have appeared to influence the work reported in this paper.

## Footnotes

1 OECD (2018), Gross domestic spending on R&D (indicator). doi: 10.1787/d8b068b4-en (Accessed on 07 August 2018) using constant 2010 US dollars.

2 Data from American Association for the Advancement of Science, based on OMB Historical Tables in Budget of the United States Government FY 2019. Constant dollar conversions based on GDP deflators, https://www.aaas.org/page/historical-trends-federal-rd.

3 Data from Statistical Abstract of the United States: 2010 (129th Edition), Table 127, National Health Expenditures–Summary, and Projections, adjusting to constant 2006 US dollars.

4 The data on S&E employment level for 1960 are calculated using the Census Bureau's 1960 Decennial Census microdata, adjusted by the Integrated Public Use Microdata Series (IPUMS) from the University of Minnesota's Minnesota Population Center (http://www.ipums.org). The data for 2011 are calculated using the 2011 American Community Survey (ACS) public use microdata sample (PUMS) files from the Census Bureau (http://www.census.gov/acs/www/data_documentation/public_use_microdata_sample/). S&E employment levels for 1960 and 2011 include workers at all education levels and do not include S&E postsecondary teachers. Although the 1960 Decennial Census data allow for separate identification of S&E postsecondary teachers, the 2011 ACS data aggregate all postsecondary teachers into one occupation code and therefore do not allow for separate identification of S&E postsecondary teachers. For 1960, including S&E postsecondary teachers would increase the number of workers employed in S&E occupations to nearly 1.2 million.

5 In clinical studies, a surrogate end-point is the measure of a biological characteristic that substitutes for a desired clinical outcome (Buyse et al., 2010). Using surrogate end-points instead of clinical end-points reduces costs and time to complete clinical trials but with the risk that the end-point does not accurately predict the desired clinical outcome.

6 Patent data available through the United States Patent and Trademark Office, U.S. Patent Statistics Chart Calendar Years 1963-2015, https://www.uspto.gov/web/offices/ac/ido/oeip/taf/us_stat.htm. STEM worker data available through the National Science Foundation data portal: https://nsf.gov/nsb/sei/edTool/data/workforce-01.html. The NSF calculated the data on S&E employment level for 1960 “using the Census Bureau's 1960 Decennial Census microdata, adjusted by the Integrated Public Use Microdata Series (IPUMS) from the University of Minnesota's Minnesota Population Center (http://www.ipums.org). The data for 2011 are calculated using the 2011 American Community Survey (ACS) public use microdata sample (PUMS) files from the Census Bureau (http://www.census.gov/acs/www/data_documentation/public_use_microdata_sample/). S&E employment levels for 1960 and 2011 include workers at all education levels and do not include S&E postsecondary teachers. Although the 1960 Decennial Census data allow for separate identification of S&E postsecondary teachers, the 2011 ACS data aggregate all postsecondary teachers into one occupation code and therefore do not allow for separate identification of S&E postsecondary teachers. For 1960, including S&E postsecondary teachers would increase the number of workers employed in S&E occupations to nearly 1.2 million.”

7 Patent data available through the United States Patent and Trademark Office, U.S. Patent Statistics Chart Calendar Years 1963-2015, https://www.uspto.gov/web/offices/ac/ido/oeip/taf/us_stat.htm. Total research expenditures are those calculated by OECD (2018), Gross domestic spending on R&D (indicator). doi: 10.1787/d8b068b4-en (Accessed on 07 August 2018).
